# A Theoretical Analysis of the Feasibility of a Singularity-Induced Micro-Electroporation System

**DOI:** 10.1371/journal.pone.0018523

**Published:** 2011-04-08

**Authors:** Gregory D. Troszak, Boris Rubinsky

**Affiliations:** 1 Department of Mechanical Engineering, University of California, Berkeley, California, United States of America; 2 Graduate Program in Biophysics and Department of Mechanical Engineering, University of California, Berkeley, California, United States of America; George Mason University, United States of America

## Abstract

Electroporation, the permeabilization of the cell membrane lipid bilayer due to a pulsed electric field, has important implications in the biotechnology, medicine, and food industries. Traditional macro and micro-electroporation devices have facing electrodes, and require significant potential differences to induce electroporation. The goal of this theoretical study is to investigate the feasibility of singularity-induced micro-electroporation; an electroporation configuration aimed at minimizing the potential differences required to induce electroporation by separating adjacent electrodes with a nanometer-scale insulator. In particular, this study aims to understand the effect of (1) insulator thickness and (2) electrode kinetics on electric field distributions in the singularity-induced micro-electroporation configuration. A non-dimensional primary current distribution model of the micro-electroporation channel shows that while increasing insulator thickness results in smaller electric field magnitudes, electroporation can still be performed with insulators thick enough to be made with microfabrication techniques. Furthermore, a secondary current distribution model of the singularity-induced micro-electroporation configuration with inert platinum electrodes and water electrolyte indicates that electrode kinetics do not inhibit charge transfer to the extent that prohibitively large potential differences are required to perform electroporation. These results indicate that singularity-induced micro-electroporation could be used to develop an electroporation system that consumes minimal power, making it suitable for remote applications such as the sterilization of water and other liquids.

## Introduction

Electroporation is the permeabilization of the cell membrane lipid bilayer due to a pulsed electric field [1]. While the physical mechanism that causes electroporation is not fully understood, it is believed that pulsed electric fields significantly increase the potential difference at the cell membrane, resulting in the formation of transient or permanent pores [2]–[6]. A review of the various theories on electroporation can be found in [7], and a comprehensive review on the thermodynamics of membrane polarization and pore formation can be found in [8]. Recent studies using molecular dynamics [9]–[11], statistical and asymptotic analysis [12], and experimental studies [13] suggest that these pores have length scales on the order of one nanometer, and start forming within nanoseconds after the application of a pulsed electric field.

Electroporation experiments show that the extent of pore formation primarily depends on the strength and duration of the pulsed electric field, causing membrane permeabilization to be reversible of irreversible [14]. Reversible electroporation is commonly used to transfer macromolecules such as proteins [15], DNA [3], [16], and drugs into cells [17], while the destructive nature of irreversible electroporation makes it suitable for sterilization [18]–[23].

In a typical electroporation procedure, a suspension of cells is placed between a pair of electrodes and a pulsed electric field is applied. While this procedure is capable of treating large quantities of cells, electroporation parameters must be determined based on the average properties of the cell population. Therefore, the extent of permeabilization varies throughout the treated cells [24]. Variations in permeabilization can be remedied by performing electroporation on individual cells, termed single cell micro-electroporation. The primary advantage of micro-electroporation is the ability to easily handle and manipulate individual cells, making it possible to control the extent of membrane permeabilization through real-time monitoring of pore formation [25], [26].

While micro-electroporation enables greater control of membrane permeabilization, generating high-strength electric fields is a challenge. Most macro and micro-electroporation devices have facing electrodes [25]. Because of this, the electric field generated between the electrodes is inversely proportional to their separation distance. Although the separation distances in micro-electroporation devices are significantly smaller than those in typical macro-electroporation devices, they are limited by cell size. Since most cells have sizes on the order of 10 microns, significant potential differences are required to induce electroporation [25].

**Figure 1:**
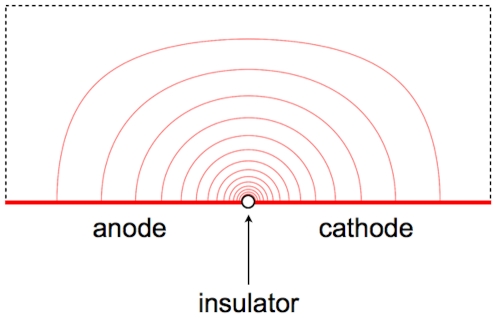
Electric field streamlines in a micro-electroporation configuration with adjacent electrodes separated by an infinitesimally small insulator. A radially-varying electric field is present.

Previously, our group conceived a micro-electroporation configuration that enables the generation of high-strength electric fields with a small potential difference. The configuration, termed singularity-induced micro-electroporation, is composed of an electrolyte atop two adjacent electrodes separated by an infinitesimally small insulator. Application of a small potential difference between the adjacent electrodes results in a radially varying electric field emanating from the infinitesimally small insulator (Fig. 1). Since it has been shown that applying an electric field along small portions of the cell membrane can induce electroporation, this radially varying electric field can be used to electroporate cells suspended in the electrolyte [27], [28].

**Figure 2:**
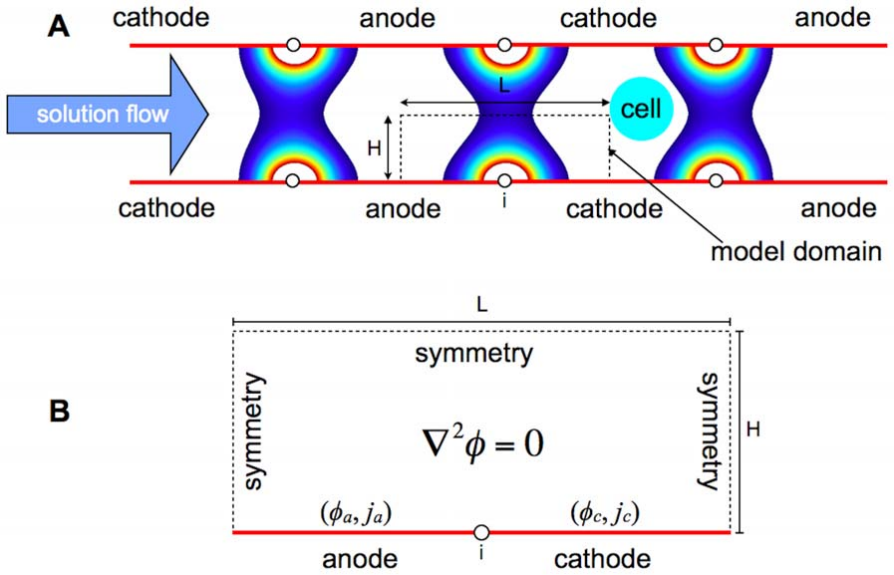
(**A**) Schematic of the micro-electroporation channel with model domain and radially-varying electric fields. Cells flowing through the micro-electroporation channel will experience a pulsed electric field, inducing electroporation. (**B**) Detailed schematic of the model domain for the primary, and secondary, current distribution models.

In our previous work, we used the singularity-induced micro-electroporation configuration to create a micro-electroporation channel. The micro-electroporation channel is formed by mirroring the singularity-induced micro-electroporation configuration and placing it in series, generating multiple electric fields (Fig. 2A). Flowing a cell suspension through the channel will cause cells to experience a pulsed electric field, inducing electroporation. A non-dimensional primary current distribution model of the micro-electroporation channel showed that decreasing channel height results in an exponential increase in the electric field magnitudes, and that cells experience exponentially greater electric field magnitudes the closer they are to the channel walls [28].

Traditional macro and micro-electroporation devices require a pulse generator and power supply. However, in the micro-electroporation channel, the need for a pulse generator is eliminated since it contains a series of adjacent electrodes. Furthermore, since the micro-electroporation channel only requires a small potential difference, electrode depletion and bubble formation, both of which adversely affect the electroporation process, can be reduced, and a minimal power source (such as a battery) is needed [25]. Additionally, reducing the potential difference required to perform electroporation enables the development of electroporation devices that utilize small power sources (such as batteries), and could potentially facilitate the creation of electroporation devices that do not require an external power source (self-powered electroporation devices). This increases the accessibility of electroporation, making its use feasible for a wide range of non-traditional applications such as the sterilization of water [29], [30], turbid beverages [31], and drugs [32].

In order to implement the micro-electroporation channel, or other devices utilizing singularity-induced micro-electroporation, the practical feasibility of the configuration needs to be further analyzed. Understanding the effect of (1) insulator thickness and (2) electrode kinetics on electric field distributions in the singularity-induced micro-electroporation configuration is particularly important.

The insulator is the smallest feature in the singularity-induced micro-electroporation configuration. Because of this, it is one of the factors limiting the implementation of devices that utilize the singularity-induced micro-electroporation configuration. In our previous work, the insulator was assumed to be infinitesimally small, which is not practically feasible. Therefore, the effect of insulator thickness on electric field distribution in the singularity-induced micro-electroporation configuration needs to be analyzed to ensure that insulators thick enough to be created with microfabrication techniques can generate electroporation inducing electric field magnitudes at small potential differences.

In order to perform singularity-induced micro-electroporation with only a minimal power source (such as a battery), a direct current must be transferred from the electrodes to the electrolyte via electrochemical reactions [33]. Because of this, the kinetics of the electrochemical reactions at the electrodes can inhibit current transfer. For singularity-induced micro-electroporation, the primary implication of inhibited current transfer is that prohibitively large potential differences could be required to generate electroporation inducing electric fields magnitudes. In order to ensure that this is not the case, the effect of electrode kinetics on electric field magnitudes in the singularity-induced micro-electroporation configuration need to be examined.

In this paper we present (1) a modified, non-dimensional, primary current distribution model to analyze the effect of insulator thickness on the micro-electroporation channel, and (2) a secondary current distribution model of the singularity-induced micro-electroporation configuration with platinum electrodes and water electrolyte. The primary purpose of these models is to further assess the feasibility of singularity-induced micro-electroporation. Additionally, the secondary current distribution model is used to investigate the effect of water conductivity and applied voltage on the electric field distribution, and power input of the singularity-induced micro-electroporation configuration.

## Methods

### 1 Modified, non-dimensional, primary current distribution model for analyzing the effect of insulator thickness on the micro-electroporation channel

Our previously developed, two-dimensional, steady-state, primary current distribution model was non-dimensionalized to analyze the effect of insulator thickness on the electric field in the electrolyte of the micro-electroporation channel (Fig. 2B) [28]. Since this model neglects surface and concentration losses at the electrode surfaces, it is governed by the Laplace equation:
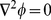
(1)where 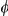 is the electric potential [33]. Furthermore, electrode surfaces are assumed to be at a constant potential, making the boundary conditions at the adjacent electrode surfaces:
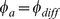
(2)

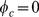
(3)where 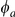 and 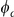 are the potentials at the anode and cathode, respectively, 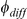 is the potential difference between the them. The remaining boundaries are insulation/symmetry boundaries and are governed by:
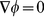
(4)Substituting the non-dimensional variables:
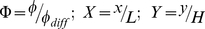
(5)into the Laplace equation in two-dimensional Cartesian coordinates yields:
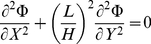
(6)


In the above relations, 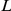 is the active electrode length and 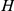 is half of the height of the micro-electroporation channel. Defining the non-dimensional geometry parameter (aspect ratio):
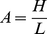
(7)the non-dimensional Laplace equation becomes:
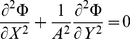
(8)Substitution of the non-dimensional variables into the boundary conditions yields:
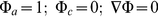
(9)Finally, the non-dimensional insulator thickness (relative insulator thickness) is defined as:
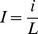
(10)where 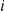 is the insulator thickness.

#### 1.1 Model solution

The non-dimensional primary current distribution model is characterized by the aspect ratio (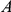) and relative insulator thickness (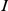). A parametric study was performed by varying 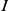 and 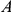 in a series of models. In each model, the non-dimensional potential distribution was solved for using a finite difference method implemented in MATLAB (R2007a version 7.4). A non-dimensional electric field defined as:
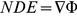
(11)was calculated using the non-dimensional potential distribution.

### 2 Secondary current distribution model of singularity-induced micro-electroporation

A two-dimensional, steady-state, secondary current distribution model was developed to analyze the effects of electrode kinetics on singularity-induced micro-electroporation. Like primary current distribution models, secondary current distribution models account for electric field effects from ohmic losses in the bulk electrolyte, and are therefore governed by the Laplace equation (Eqn. 1) in that region. However, unlike primary current distribution models, secondary current distribution models account for kinetic losses at the electrode surfaces [33]. Since kinetic losses strongly depend on the potential at an electrode surface, the boundary conditions at the adjacent electrode surfaces are:
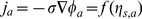
(12)

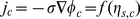
(13)where 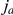 and 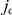 are the current densities at the anode and cathode, respectively, 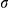 is the conductivity of the bulk electrolyte, and 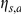 and 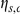 are the surface overpotentials at the anode and cathode, respectively. Overpotential represents a departure from the equilibrium potential at an electrode surface, and is defined as:
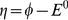
(14)where 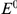 is the equilibrium potential for an electrochemical reaction at standard state, typically 293 K at 1 atm [33].

#### 2.1 Electrode kinetics model

Neglecting concentration losses, the relationship between current and potential at electrode surfaces is commonly described by a modified version of the Butler-Volmer model [34]:
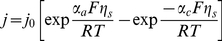
(15)


Conceptually, the first term describes the anodic (reduction) contribution to the net current at a given potential, while the second term describes the cathodic (oxidation) contribution to the net current. With that in mind, the variables in the Butler-Volmer model are:

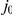
, the exchange current density. The exchange current density is the current density where the anodic and cathodic contributions are equal, resulting in no net current.

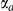
 and 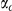, the anodic and cathodic transfer coefficients, which respectively describe the energy required for each reaction to occur.

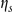
, the surface overpotential, the deviation of the electrode potential from its equilibrium potential.

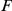
, the Faraday constant (96500 C/mol).

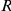
, the universal gas constant (8.314 J/mol-K).

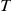
, the temperature of the electrode reaction (K).

The exchange current density, and the anodic and cathodic transfer coefficients are determined experimentally, typically by fitting current-potential data to the Butler-Volmer model [34]. However, in some cases, it is more convenient to fit current-potential data to simpler forms (i.e. linear) [34].

#### 2.2 Development of the current density boundary conditions

A voltage must be applied to the cell suspension to generate an electric field for electroporation. Because of potential losses due to irreversibilities (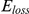), the applied voltage (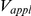) must be greater than the equilibrium potential (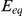) of the electrochemical cell [33]:
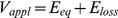
(16)


The equilibrium potential of the electrochemical cell is the difference between the anode and cathode reduction equilibrium potentials at standard state (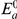 and 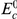, respectively) [33], [34]:
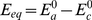
(17)


Irreversible losses have three classifications [33], [34]:

1. Surface losses from sluggish electrode kinetics.
2. Concentration losses due to mass-transfer limitations.
3. Ohmic losses in the electrolyte.

Since concentration losses are neglected in secondary current distribution models, the irreversible losses can be represented as:
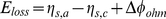
(18)where 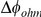 is the ohmic loss in the electrolyte, and can be further decomposed to:
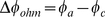
(19)Combining Eqns. 17, 18, and 19:
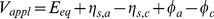
(20)provides a more detailed relation for the voltage that must be applied to the electrochemical cell to compensate for irreversible losses. Since kinetic models provide the net current density at an electrode surface as a function of surface overpotential, the equation above can be separated to obtain the surface overpotentials at the anode and cathode:
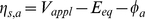
(21)

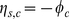
(22)Substituting these relations into the modified version of the Butler-Volmer equation relates the surface potentials at the anode and cathode to their respective current densities, enabling an implicit numerical solution.
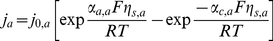
(23)

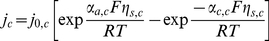
(24)


#### 2.3 Model parameters

The parameters used in the secondary current distribution model are outlined in Table 1.

**Table 1:** Secondary current distribution model parameters.

Global			
Faraday constant	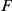	C mol∧−1	96500
Universal gas constant	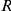	J mol∧−1 K∧−1	8.314
Temperature	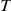	K	298
Electrochemical cell equilibrium potential	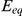	V	1.23
Applied voltage	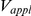	V	1.3–2.5
Water conductivity	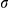	S m∧−1	0.0005, 0.005, 0.05

The secondary current distribution model domain is shown in Fig. 2B. The domain is 10 microns long, has a 100 nanometer thick insulator, and is 20 microns high. Since previous results show that decreasing domain height exponentially increases electric field magnitudes, the height of the domain was made sufficiently large to determine the minimum electric field magnitudes that can be generated when accounting for electrode kinetics [28].

Since we would like to use the singularity-induced micro-electroporation configuration for water sterilization, the bulk electrolyte is water. The electrical conductivity of water typically varies between 0.0005 and 0.05 S/m [35].

The anode and cathode are modeled as inert platinum electrodes. In water, the electrochemical reactions that take place at the electrode surfaces are identical to those in water electrolysis [36]. At the anode, water is oxidized:
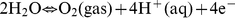
(25)Under standard conditions, this reaction has a reduction equilibrium potential (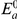) of 1.23 V and an exchange current density (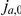) of 10^−8^ A/m^2^ [33]. Additionally, the transfer coefficients (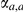 and 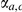) were assumed to be 0.5 [34]. At the cathode, water is reduced:
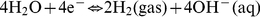
(26)Under standard conditions, this reaction has a reduction potential (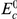) of −0.83 V and an exchange current density (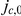) of 10 A/m^2^ [33]. Similar to the water oxidation reaction at the anode, the transfer coefficients (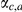 and 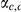) were assumed to be 0.5 [34]. Therefore, the net reaction in the platinum-water singularity-induced micro-electroporation system is:
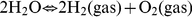
(27)Under standard conditions, this reaction has an equilibrium potential (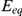) of 2.06 V that must be exceeded to generate an electric field distribution in the water.

It should be noted that since saline is a water based solution, these electrochemical reactions are also applicable to a more traditional electroporation system. Therefore, this secondary current distribution model could easily be modified to analyze singularity-induced micro-electroporation in a saline solution by changing the bulk electrolyte conductivity.

#### 2.4 Model solution

The secondary current distribution model is affected by the conductivity of the water electrolyte (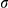) and voltage applied (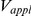) to the electrochemical cell. A parametric study was performed by varying these parameters in a series of models. In each model, the potential distribution was solved for using the finite element analysis software COMSOL Multiphysics 4.0a. The electric field defined as:
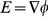
(28)was calculated using the potential distribution. Furthermore, by integrating the current density at the anode or cathode boundary, the total current (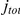) through the model was determined. Using the total current through the model, the power input defined as:
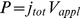
(29)was calculated.

## Results

### 1 Non-dimensional primary current distribution model for analyzing the effect of insulator thickness

**Figure 3:**
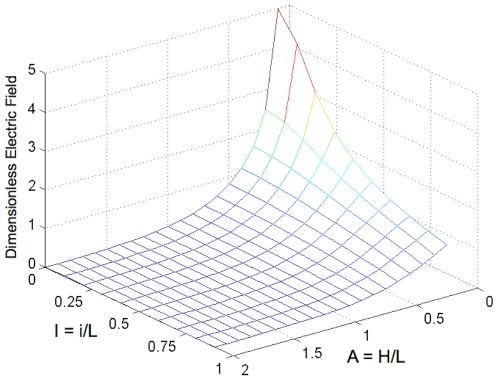
Non-dimensional electric field (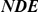) magnitudes at X = 0.5, Y = 1 for various relative insulator thicknesses (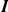) and domain aspect ratios (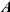). At low aspect ratios, decreasing the relative insulator thickness substantially increases non-dimensional electric field magnitude. At high aspect ratios, decreasing the relative insulator thickness negligibly influences non-dimensional electric field magnitude.

The results of the non-dimensional primary current distribution model show that decreasing the relative insulator thickness (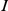) increases the magnitude of the non-dimensional electric field (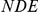) at the center of the micro-electroporation channel (Fig. 3). More specifically, the extent of the increase in the non-dimensional electric field magnitude due to relative insulator thickness depends on the aspect ratio (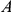). At low aspect ratios, decreasing relative insulator thickness substantially increases the non-dimensional electric field. Decreasing the relative insulator thickness from 0.9 to 0 (singularity) at an aspect ratio of 0.1 results in a 413% increase in non-dimensional electric field magnitude. Conversely, at high aspect ratios, decreasing the relative insulator thickness negligibly increases the non-dimensional electric field. At an aspect ratio of 2, decreasing the relative insulator thickness from 0.9 to 0 results in a 115% increase in non-dimensional electric field magnitude.

### 2 Secondary current distribution model of singularity-induced micro-electroporation

#### 2.1 Effect of water conductivity and applied voltage on electric field distribution

**Figure 4:**
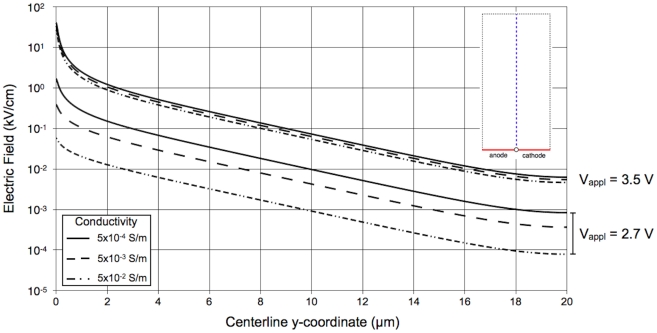
Electric field magnitudes along a centerline directly above the insulator (shown in upper-right corner) in the secondary current distribution model. At applied voltage lower than ∼3.2 V, conductivity substantially influences electric field magnitudes and increases in applied voltage increase electric field magnitudes. At applied voltages higher than ∼3.2 V, conductivity negligibly influences electric field magnitudes and increases in applied voltage do not affect electric field magnitudes.

The conductivity of the water (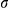) and the applied voltage (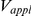) both influence the electric field distribution in the singularity-induced micro-electroporation configuration. At applied voltages lower than ∼3.2 V, low conductivity water contains substantially larger electric field magnitudes than high conductivity water (Fig. 4). For example, at an applied voltage of 2.7 V, the electric field magnitudes at the center of the insulator are 0.06, 0.38, and 1.64 kV/cm at water conductivities of 0.05, 0.005, and 0.0005 S/m, respectively. Furthermore, at applied voltages lower than 2.8 V, increasing the applied voltage exponentially increases electric field magnitudes in the water. Conversely, at applied voltages higher than 2.8 V, the electric field distribution becomes constant and independent of water conductivity. At an applied voltage of 3.5 V, the electric field magnitudes at the center of the insulator are 26.4, 33.1, and 39.8 kV/cm at water conductivities of 0.05, 0.005, and 0.0005 S/m, respectively.

#### 2.2 Effect of water conductivity and applied voltage on power input

**Figure 5:**
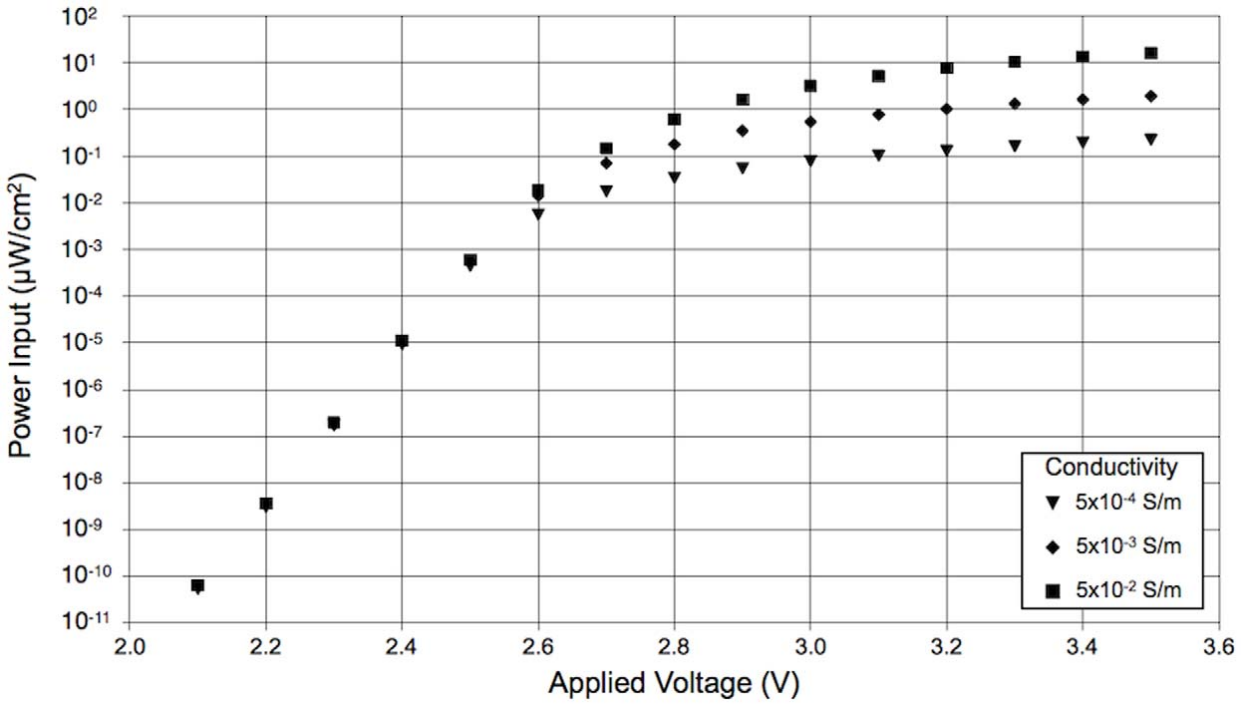
Power input to the singularity-induced micro-electroporation configuration depends on applied voltage and water conductivity. At low applied voltages, conductivity negligibly affects power input and increases in applied voltage exponentially increase power input. At high applied voltages, low conductivity water requires the least power input and increases in applied voltage negligibly affect power input.

The power input to the singularity-induced micro-electroporation configuration is also dependent on the conductivity of the water and the applied voltage (Fig. 5). At applied voltages less than ∼2.6 V, power input is independent of water conductivity and increases exponentially with applied voltage. For example, at an applied voltage of 2.4 V, the powers input to the singularity-induced micro-electroporation configuration are 1.09, 1.05, and 0.92×10^−5^ µW/cm^2^ at water conductivities of 0.05, 0.005, and 0.0005 S/m, respectively. Conversely, at applied voltages greater than ∼2.6 V, the power input becomes constant and is highly dependent on the water conductivity. A singularity-induced micro-electroporation configuration with low conductivity water (0.0005 S/m) requires the least power input, 0.23 µW/cm^2^ at an applied voltage of 3.5 V. The power input required by the singularity-induced micro-electroporation configuration substantially increases with water conductivity. Configurations with 0.005 and 0.05 S/m water conductivities require 1.93 and 16.20 µW/cm^2^, respectively.

## Discussion

### 1 Effect of insulator thickness

The results of the non-dimensional primary current distribution model demonstrate the practical feasibility of the micro-electroporation channel. In our previous work, we predicted that increasing the insulator thickness would decrease the electric field magnitudes throughout the electrolyte of the micro-electroporation channel [28]. While our results quantitatively support this prediction, they also indicate that electroporation inducing electric fields can be generated with insulators thick enough to be created with microfabrication techniques. For example, applying a 0.5 V potential difference in a micro-electroporation channel with an active electrode length (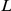) of 10 µm, micro-electroporation channel height (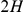) of 2 µm, and insulator thickness (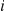) of 100 nm (non-dimensional data for 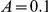, 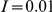), can generate electric field magnitudes in excess of 10 kV/cm, which are sufficient for inducing irreversible electroporation [21]. Numerous lithographic techniques are capable of producing sub-100 nm features, and could be used to create the insulators in a micro-electroporation channel. Immersion lithography is a photolithography enhancement technique that places a liquid with a refractive index greater than one between the final lens and wafer. Current immersion lithography tools are capable of creating feature sizes below 45 nm [37]. Additionally, electron beam lithography, a form of lithography that uses a traveling beam of electrons, can create features smaller than 10 nm [38].

### 2 Secondary current distribution model of singularity-induced micro-electroporation

Electrochemical reactions must transfer a direct current from the electrodes to the electrolyte to perform singularity-induced micro-electroporation. The kinetics of electrochemical reactions can inhibit current transfer and potentially necessitate prohibitively large potential differences to generate electroporation-inducing electric field magnitudes. Therefore, to adequately analyze the feasibility of implementing a singularity-induced micro-electroporation system, the effect of electrode kinetics on electric field magnitudes must be understood. The secondary current distribution model of the singularity-induced micro-electroporation configuration with platinum electrodes and water electrolyte accounts for electrode kinetics. The results of this model: (1) demonstrate the practical feasibility of implementing a singularity-induced micro-electroporation system, (2) predicts the upper limit to the electric field magnitudes of the system, and (3) provides data for optimizing the power input necessary to obtain a desired electric field distribution.

The practical feasibility of creating a singularity-induced micro-electroporation system is demonstrated by the results of the secondary current distribution model with platinum electrodes and water electrolyte. The results show that electric fields in excess of those required to induce reversible (1–3 kV/cm) and irreversible (10 kV/cm) electroporation can be generated in water with platinum electrodes [21]. For instance, in water with a conductivity of 0.0005 S/m, an applied voltage as low as 2.8 V (0.7 V larger than 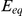) can generate electric fields sufficient to induce reversible electroporation near the insulator surface. Increasing the applied voltage by 0.1 V generates electric fields capable of inducing irreversible electroporation near the insulator surface, and reversible electroporation at distances up to ∼0.7 µm from the insulator. Although lower electric field magnitudes are present in higher conductivity water (0.005 or 0.05 S/m), minor increases in applied voltage result in similar reversible and irreversible electroporation inducing electric fields.

The trend shown in Fig. 4 indicates that there is an upper limit to the electric field magnitudes that can be generated in the singularity-induced micro-electroporation system. For this system, the low exchange current density of the anode electrochemical reaction (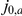) limits the current through the system. As a result, as the applied voltage increases, the water conductivity has less of an influence on the electric field distribution. Furthermore, at large applied voltages, increasing the applied voltage negligibly changes the electric field distribution, indicating the upper limit of the electric field magnitudes that can be generated with this system. Close to the insulator, the electric field magnitudes at the upper limit are well above the magnitudes required to induce reversible and irreversible electroporation. However, if large electric field magnitudes are required away from the insulator, the upper limit may become an important design consideration.

The secondary current distribution model of singularity-induced micro-electroporation can be used to optimize the power input to the system. As previously noted, at large applied voltages, water conductivity is negligibly influential and the electric field distribution becomes constant with increasing applied voltage (Fig. 4). Fig. 5 shows that while power input also becomes constant at large applied voltages, it is substantially affected by water conductivity. In general, low conductivity water (0.0005 S/m) generates the largest electric field magnitudes with the least power input, and high conductivity water (0.05 S/m) generates the smallest electric field magnitudes with the most power input. Therefore, decreasing the water conductivity is the most effective method for optimizing the power input to the system.

It should be noted that the methodology used for developing the secondary current distribution model of singularity-induced micro-electroporation could be used to model a variety of electroporation devices. With appropriate electrode kinetics parameters, numerous electrode materials and electroporation configurations could be examined. These models would aid in experimental studies by providing electric field distributions throughout the electrolyte. Additionally, they would facilitate the optimal design of electroporation systems for a variety of applications.

The singularity-induced micro-electroporation configuration offers numerous advantages over traditional macro and micro-electroporation devices. In electroporation devices with facing electrodes, a cell's proximity has no bearing on the electric field magnitude it will experience. Conversely, in a singularity-induced micro-electroporation configuration, the electric field magnitude experienced by a cell is dictated by the gap between the cell and the surface of the configuration. Because of this, cell size does not affect the potential difference required to achieve a desired electric field.

Another advantage of the singularity-induced micro-electroporation configuration over traditional macro and micro-electroporation devices is that less electrical equipment is required. Traditional macro and micro-electroporation devices require a pulse generator and power supply. However, by placing singularity-induced micro-electroporation configurations in series, as is done in the micro-electroporation channel, the need for a pulse generator is eliminated. Furthermore, as validated by the secondary current distribution model, only a small potential difference is required. Because of this, only a minimal power source (such as a battery) is needed.

### 3 Conclusions

The practical feasibility of singularity-induced micro-electroporation systems were assessed by examining the effect of insulator thickness and electrode kinetics on generated electric field distributions. Two models were developed to understand these effects: (1) a modified, non-dimensional, primary current distribution model of a micro-electroporation channel, and (2) a secondary current distribution model of the singularity-induced micro-electroporation configuration with platinum electrodes and water electrolyte.

A previously developed, non-dimensional, primary current distribution model was modified to analyze the effect of insulator thickness on the electric field distribution of a micro-electroporation channel. Increasing the insulator thickness exponentially reduces the electric field magnitude directly above the center of the insulator and inhibits the permeation of high-strength electric fields in the electrolyte. However, high-strength electric fields can still be generated with insulators thick enough to be created with MEMS manufacturing techniques [37], [38]. Therefore, insulator thickness does not inhibit the practical feasibility of creating singularity-induced micro-electroporation systems.

A secondary current distribution model of the singularity-induced micro-electroporation configuration with platinum electrodes and water electrolyte was developed to examine the effect of electrode kinetics on the electric field distribution in the water. The results of this model show that electric field magnitudes in excess of those required to induce reversible (1–3 kV/cm) and irreversible (10 kV/cm) electroporation can be generated in water with platinum electrodes [21]. This further substantiates the practical feasibility of implementing a singularity-induced micro-electroporation device. Additionally, the secondary current distribution model shows that at low applied voltages, significantly larger electric field magnitudes are present in lower conductivity water. Initially, as the applied voltage increases there is an exponential increase in electric field magnitudes in the water. However, at large applied voltages, increasing the applied voltage negligibly changes the electric field magnitudes, regardless of water conductivity. Furthermore, at large applied voltages, the required power input is highly dependent on the conductivity of the water. Therefore, it can be concluded that low conductivity water generates the largest electric field magnitudes with the least power input, and high conductivity water generates the smallest electric field magnitudes with the most power input.

Although a great deal of work needs to be done to bring singularity-induced micro-electroporation to fruition, this theoretical study indicates that pursing that work is worthwhile. The simplicity of electroporation makes it a powerful technology. Devices implementing the singularity-induced micro-electroporation configuration increase the accessibility of electroporation, making its use feasible for a wide range of non-traditional applications.

## References

[1] Weaver JC, Chizmadzhev YA (1996). Theory of electroporation: a review.. Bioelectrochem Bioenerg.

[2] Wong T, Neumann E (1982). Electric field mediated gene transfer.. Biochem Biophys Res Commun.

[3] Neumann E, Schaeffer-Ridder M, Wang Y, Hofschneider PH (1982). Gene transfer into mouse lymphoma cells by electroporation in high electric fields.. EMBO J.

[4] Sugar IP, Förster W, Neumann E (1987). Model of cell electrofusion: membrane electroporation, pore coalescence and percolation.. Biophys Chem.

[5] Zimmerman U, Pilwat G, Rieman F (1974). Dielectric breakdown of cell membranes.. Biophys J.

[6] Abidor IG, Arakelyan VB, Chernomordik LV, Chizmadzev YA, Ptsushenko VF (1979). Electric breakdown of bilayer lipid membranes: I. The main experimental facts and their qualitative discussion.. J Electroanal Chem Interfacial Electrochem.

[7] Chen C, Smye SW, Robinson MP, Evans JA (2006). Membrane electroporation theories: a review.. Med Biol Eng Comput.

[8] Newman E, Kakorin S, Pkhomov AG, Miklavcic D, Markov MS (2010). Physical chemical theory of membrane electroporation and electrotransfer of biogenic agents.. Advanced electroporation techniques in biology and medicine.

[9] Tieleman DP, Leontiadou H, Mark AE, Marrink SJ (2003). Simulation of pore formation in lipid bilayers by mechanical stress and electric fields.. J Am Chem Soc.

[10] Tarek M (2005). Membrane electroporation: a molecular dynamics simulation.. Biophys J.

[11] Vernier PT, Ziegler MJ, Sun Y, Chang WV, Gundersen MA (2006). Nanopore formation and phophatidylserin externalization in a phospholipid bilayer at high transmembrane potential.. J Am Chem Soc.

[12] Krassowska Neu W, Neu JC, Rubinsky B (2010). Mechanism of irreversible electroporation in cells: Insight from the models.. Irreversible electroporation.

[13] Pakhomov AG, Bowman AM, Ibey BL, Andre FM, Pakhomova ON (2009). Lipid nanopores can form a stable, ion channel-like conduction pathway in cell membrane.. Biochem Biophys Res Comm.

[14] Ho SY, Mittal GS (1996). Electroporation of cell membranes: A review.. Crit Rev Biotechnol.

[15] Ho SY, Mitall GS, Cross JD (1997). Effects of high field electric pulses on the activity of selected enzymes.. J Food Eng.

[16] Prasanna GL, Panda T (1997). Electroporation: basic principles, practical considerations and applications in molecular biology.. Bioprocess Biosyst Eng.

[17] Tsong TY, Kinosita K (1985). Use of voltage pulses for the pore opening and drug loading, and the subsequent resealing of red blood cells.. Bibl Haematol.

[18] Knorr D, Angersbach A, Eshtiaghi MN, Heinz V, Lee D (2001). Processing concepts based on high intensity electric field pulses.. Trends Food Sci Tech.

[19] Mastwijk HC, Bartels PV (2004). Pulsed electric field processing in the fruit juice and dairy industries.. Int Rev Food Sci Technol.

[20] Wouters PC, Dutreux N, Smelt JPPM, Lelieveld HLM (1999). Effects of pulsed electric fields on inactivation kinetics of Listeria innocua.. Appl Environ Microbiol.

[21] Sale AJH, Hamilton WA (1967). Effects of high electric fields on microorganisms: I. Killing of bacteria and yeasts.. Biochim Biophys Acta.

[22] Hamilton WA, Sale AJH (1967). Effects of high electric fields on microorganisms: II. Mechanism of action of the lethal effect.. Biochim Biophys Acta.

[23] Sale AJH, Hamilton WA (1968). Effects of high electric fields on microorganisms: III. Lysis of erythrocytes and protoplasts.. Biochim Biophys Acta.

[24] Andreason GL (1993). Electroporation as a technique for the transfer of macromolecules into mammalian cell lines.. J Tiss Cult Meth.

[25] Fox MB, Esveld DC, Valero A, Luttge R, Mastwijk HC (2006). Electroporation of cells in microfluidic devices: a review.. Anal Bioanal Chem.

[26] Huang Y, Rubinsky B (1999). Micro-electroporation: improving the efficiency and understanding of electrical permeabilization of cells.. Biomed Microdevices.

[27] Diaz-Rivera RE, Rubinsky B (2006). Electrical and thermal characterization of nanochannels between a cell and a silicon based micro-pore.. Biomed Microdevices.

[28] Troszak GD, Rubinsky B (2010). A primary current distribution model of a novel micro-electroporation channel configuration.. Biomed Microdevices.

[29] World Health Organization/United Nations Children's Fund, Water Supply and Sanitation Collaborative Council (2000). Global water supply and sanitation assessment report.. http://www.who.int/docstore/water_sanitation_health/Globassessment/GlobalTOC.htm.

[30] World Health Organization (2009). Diarrhoeal diseases.. http://www.who.int/vaccine_research/diseases/diarrhoeal/en/print.html.

[31] Golberg A, Kandel J, Belkin M, Rubinsky B (2010). Intermittently delivered pulsed electric fields for sterile storage of turbid media.. IEEE Trans Plasma Sci.

[32] Golberg A, Belkin M, Rubinsky B (2009). Irreversible electroporation for microbial control of drugs in solution.. AAPS PharmSciTech.

[33] Prentice G (1991). Electrochemical engineering principles, 1^st^ edn.

[34] Bard AJ, Faulkner LR (2001). Electrochemical methods: fundamentals and applications, 2^nd^ edn.

[35] De Zuane J (1997). Handbook of drinking water quality, 2^nd^ edn.

[36] Saulis G, Lape R, Praneviciute R, Mickevicius D (2005). Changes of the solution pH due to exposure by high-voltage electric pulses.. Bioelectrochemistry.

[37] Owa S, Nagasaka H (2008). Immersion lithography: its history, current status and future prospects.. Proc of SPIE.

[38] Broers AN, Hoole ACF, Ryan JM (1996). Electron beam lithography-resolution limits.. Microelectron Eng.

